# Qual Escore de Risco Melhor Avalia os Objetivos Clínicos para meu Paciente com Miocardiopatia Hipertrófica?

**DOI:** 10.36660/abc.20200543

**Published:** 2020-08-19

**Authors:** Eduardo Alberto de Castro Roque

**Affiliations:** 1 Hospital Metropolitano Serra ES Brasil Hospital Metropolitano,Serra, ES - Brasil

**Keywords:** Cardiomiopatia Hipertrófica Familiar, Programa de Vigilância de Riscos, Estenose Subaórtica Fixa, Desfibriladores Implantáveis, Hipertrofia Ventricular Esquerda

A cardiomiopatia hipertrófica (CMH) é um distúrbio genético caracterizada pela hipertrofia inexplicada do ventrículo esquerdo, comumente assimétrico com maiores espessamentos do septo interventricular basal. A obstrução da via de saída do ventrículo esquerdo está presente em repouso em cerca de um terço dos pacientes e pode ser provocada em outro terço. As características histológicas da CMH incluem hipertrofia e desordem dos miócitos, além de fibrose intersticial. A hipertrofia também é frequentemente associada à disfunção diastólica do ventrículo esquerdo.

O primeiro caso de CMH foi descrito por Henri Liouville em 1869 no Gazette Medecine Paris. Em 1907, o Dr. A. Schmincke, patologista alemão, descreveu dois corações com hipertrofia ventricular esquerda; ambos vieram de mulheres na casa dos cinquenta anos. Levy e von Glahn, em 1944, da Universidade da Colômbia, em Nova York, publicaram uma série de casos que se assemelham a CMH. Em 1949, William Evans, um cardiologista de Londres, descreveu a ocorrência familiar de hipertrofia cardíaca em uma série de pacientes semelhantes aos descritos no artigo de Levy e von Glahn. Dr. Eugene Braunwald e Dr. Andrew Glenn Morrow publicaram uma série de trabalhos onde detalharam aspectos clínicos e hemodinâmicos desta doença permitindo estabelecer objetivos terapêuticos.^1 - 5^

Na maioria dos pacientes a CMH tem um curso relativamente benigno. Porém a CMH também é uma causa importante de morte súbita (MS), particularmente em adolescentes e adultos jovens, com risco de 0,5 a 2% ao ano, sendo a causa mais frequente de MS em adolescentes.^6 , 7^ A prevenção de eventos de MS através de implante de um dispositivo de implantado parece óbvia, uma vez que as opções de tratamento clínico para prevenção de arritmia grave fatal não podem ser oferecida pelo tratamento farmacológico de forma confiável. Como uma terapia de elevado custo e não isenta de eventos adversos (infecção e choque inapropriado) tornou-se mandatório estabelecer quais grupos teriam benefício com a indicação de terapia com desfibrilador implantado.

Entre os escores de avaliação de risco de MS para definir os pacientes de maior benefício, ganharam destaques a calculadora de risco da AHA publicada em 2011^8^ e a calculadora de risco para avaliação de MS pela Sociedade Europeia de Cardiologia em 2015.^9^ Com metodologias diferentes ambas se propunham auxiliar o clínico a identificar um grupo de pacientes de maior benefício.

Um classificador ideal deveria ser simples, com poucos critérios, cada critério sendo de fácil interpretação, reproduzível com base na prática existente, altamente sensível, de alto valor preditivo negativo, capaz de reduzir riscos com menor custo possível. Ainda que atenda a todos os quesitos acima, temos que levar em consideração que os modelos prognósticos são desenvolvidos para serem aplicados em novos pacientes, que podem vir de diferentes centros, de diferentes raças, hábitos, morbidades distintas e com os mais diferentes microbiomas. Portanto, novos pacientes são comumente referidos como diferentes, mas semelhantes aos pacientes usados para desenvolver os modelos. Quando uma nova população de pacientes pode ser considerada semelhante (suficiente) à população em desenvolvimento para justificar a validação e, eventualmente, a aplicação de um modelo? A resposta a esta pergunta é totalmente dependente de registros médicos que possam revalidar ferramentas que usamos em nossa prática.^10^

O trabalho em questão^11^ vai além e avalia de forma prospectiva uma coorte de pacientes aplicando as ferramentas mais usadas na atualidade para validar qual delas seria de maior acurácia em nossa população, assim como identifica pontos fortes e fragilidades de nossa capacidade de avaliar e predizer eventos futuros. A importância do trabalho vai além do tema, uma vez que demonstra a necessidade de obtermos registros, permitindo que a comunidade cientifica seja capaz de revalidar os mais distintos escores clínicos em nossa prática. Com enorme impacto em eficácia (ao reduzir ocorrência de mortes súbitas) assim como de eficiência (ao permitir que possamos alocar recursos para contemplar os pacientes com maior chance de benefício).
