# Supplementary material for: Qual Escore de Risco Melhor Avalia os Objetivos Clínicos para meu Paciente com Miocardiopatia Hipertrófica?
Source: Arq Bras Cardiol. 2020 Aug 19;115(2):205–6. [Article in Portuguese] doi: 10.36660/abc.20200543 (PMC8384274; doi:10.36660/abc.20200543)
Supplement: Translation

# Translation: Which Risk Score Best Assesses Clinical Objectives in Patients with Hypertrophic Cardiomyopathy?

**Authors:** Eduardo Alberto de Castro Roque

**Affiliations:** 1 Brazil Hospital Metropolitano, Serra, ES - Brazil

**Keywords:** Cardiomyopathy, Hypertrophic Familial, Safety Management, Discrete Subaortic Stenosis, Defibrillators Implantable, Hypertrophy, Left Ventricular

Hypertrophic cardiomyopathy (HCM) is a genetic disorder characterized by commonly asymmetric unexplained left ventricular hypertrophy, with greater thickening of the basal interventricular septum. Left ventricular outflow tract obstruction is present at rest in around one third of patients and can be caused in another third. The histological features of HCM include hypertrophy and myocyte disorder, in addition to interstitial fibrosis. Hypertrophy is also often associated with left ventricular diastolic dysfunction.

The first case of HCM was described by Henri Liouville in 1869 in the Gazette Medecine Paris. In 1907, Dr. A. Schmincke, a German pathologist, described two hearts with left ventricular hypertrophy; both were seen in women in their fifth decade of life. Levy and von Glahn, in 1944, from the University of Colombia, in New York, published a series of cases that resembled HCM. In 1949, William Evans, a cardiologist from London, described the familial occurrence of cardiac hypertrophy in a series of patients similar to those described in the article by Levy and von Glahn. Dr. Eugene Braunwald and Dr. Andrew Glenn Morrow published a series of studies where they detailed the clinical and hemodynamic aspects of this disease, allowing the establishment of therapeutic objectives.^1 - 5^

HCM has a relatively benign course in the majority of patients. However, HCM is also an important cause of sudden death (SD), particularly in adolescents and young adults, with a risk of 0.5 to 2% per year, being the most frequent cause of SD in adolescents.^6 , 7^ The prevention of SD events through a device implantation seem obvious, since the clinical treatment options for the prevention of severe fatal arrhythmia cannot be reliably offered by pharmacological treatment. As a high-cost therapy and one that is not exempt from adverse events (infection and inappropriate shock), it became mandatory to establish which groups would benefit from the indication of therapy with an implanted defibrillator.

Among the SD risk assessment scores to define which patients would have the greatest benefit, the AHA risk calculator published in 2011^8^ and the risk calculator for assessing SD by the European Society of Cardiology in 2015 were highlighted.^9^ With different methodologies, both proposed to assist the clinician in identifying a group of patients that would have the greatest benefit.

An ideal classification system should be simple, have few criteria, with each criterion being easy to interpret, be reproducible based on existing practice, highly sensitive, with high negative predictive value, capable of reducing risks at the lowest possible cost. Although it meets all the above requirements, we have to consider that the prognostic models are developed to be applied to new patients, who may come from different centers, have different ethnicities, habits, different morbidities and with the most different microbiomes. Therefore, new patients are commonly referred to as different, but similar to the patients who were used to develop the models. When can a new population of patients be considered (sufficiently) similar to the developing population to justify the validation and, possibly, the application of a model? The answer to this question is totally dependent on medical records that can revalidate tools used in our practice.^10^

The present study^11^ goes further and prospectively evaluates a cohort of patients using the tools most frequently used at present to validate which of them would be the most accurate in our population, and also identifies the strengths and weaknesses of our capacity to assess and predict future events. The importance of the study goes beyond the topic, since it demonstrates the need to obtain records, allowing the scientific community to be able to revalidate the most distinct clinical scores in our practice, with a huge impact on efficacy (by reducing the occurrence of sudden deaths) as well as efficiency (by allowing us to allocate resources to include patients with the greatest chance of benefit).
